# Excessive Mechanotransduction in Sensory Neurons Causes Joint Contractures

**DOI:** 10.1126/science.add3598

**Published:** 2023-01-12

**Authors:** Shang Ma, Adrienne E. Dubin, Luis O. Romero, Meaghan Loud, Alexandra Salazar, Sarah Chu, Nikola Klier, Sameer Masri, Yunxiao Zhang, Yu Wang, Alex T. Chesler, Katherine A. Wilkinson, Valeria Vásquez, Kara L. Marshall, Ardem Patapoutian

**Affiliations:** 1. Howard Hughes Medical Institute, Department of Neuroscience, Dorris Neuroscience Center, Scripps Research, La Jolla, CA 92037, USA; 2. Department of Physiology, College of Medicine, University of Tennessee Health Science Center, Memphis, TN, USA.; 3. Integrated Biomedical Sciences Graduate Program, College of Graduate Health Sciences, University of Tennessee Health Science Center, Memphis, TN, USA; 4. Department of Biological Sciences, San Jose State University, San Jose, CA, USA.; 5. National Institute of Neurological Disorders and Stroke, National Institutes of Health, Bethesda, MD, USA.; 6. National Center for Complementary and Integrative Health, National Institutes of Health, Bethesda, MD, USA.; 7. Department of Neuroscience, Baylor College of Medicine, Houston, TX, USA.

## Abstract

Distal arthrogryposis (DA) is a collection of rare disorders characterized by congenital joint contractures. Most DA mutations are in muscle- and joint-related genes, and the anatomical defects originate cell-autonomously within the musculoskeletal system. However, gain-of-function (GOF) mutations in PIEZO2, a principal mechanosensor in somatosensation, cause DA subtype 5 via unknown mechanisms. We show that expression of a GOF PIEZO2 mutation in proprioceptive sensory neurons mainly innervating muscle spindles and tendons is sufficient to induce DA5-like phenotypes in mice. Overactive PIEZO2 causes anatomical defects via increased activity within the peripheral nervous system during postnatal development. Further, Botox and a dietary fatty acids that modulate PIEZO2 activity reduce DA5-like deficits. This reveals a role for somatosensory neurons: excessive mechanosensation within these neurons disrupts musculoskeletal development.

## One-sentence summary

Overactive PIEZO2 in proprioceptive neurons disrupts musculoskeletal development.

Distal Arthrogryposis (DA) is a rare disorder with congenital contractures primarily affecting joints. It is estimated to afflict ~1 in 3,000 individuals worldwide and usually requires invasive surgeries to alleviate the symptoms (1, 2). Various mutations have been identified in DA patients in genes important for musculoskeletal function (3). DA5 is an autosomal dominant disorder with distinct clinical features including ophthalmoplegia and restrictive lung disease, in addition to contractures in distal joints (4). Gain-of-function (GOF) mutations in PIEZO2, a mechanically activated ion channel, have been found in DA5 patients (5). PIEZO2 is the principal mechanosensor in somatosensory neurons and underlies touch sensation (6) and proprioception (the sense of where one’s limbs are in space) (7), as well as mechanosensory processes in internal organs such as lung, aorta, and bladder (8–10). However, direct role for this ion channel in muscles or tendons has not been described.

## Overactive PIEZO2 increases mechanosensitivity of sensory neurons.

**Figure 1.**
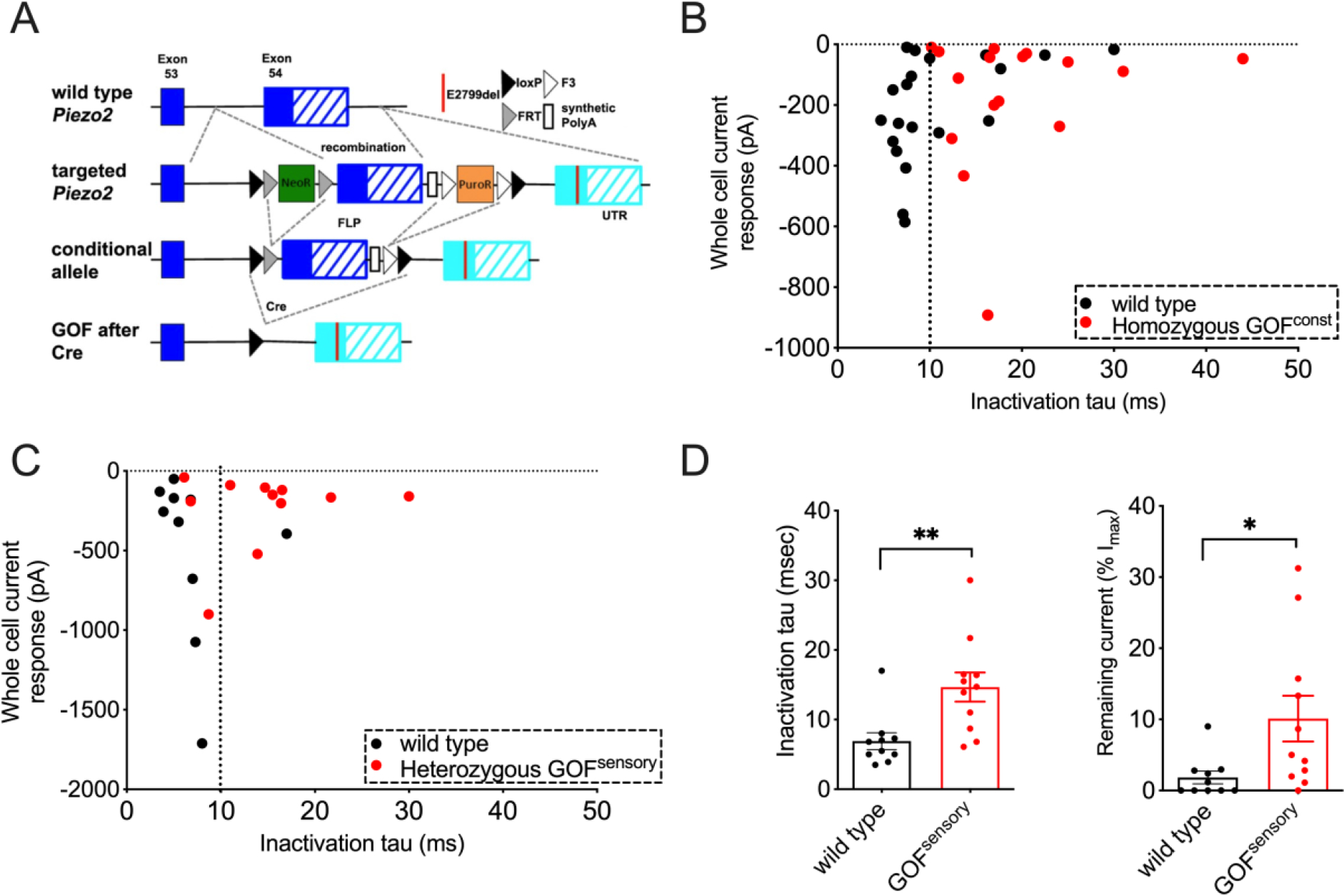
Gain-of-function (GOF) PIEZO2 increases mechanosensitivity of sensory neurons. (A) Conditional GOF *Piezo2* mouse model. Cre-dependent replacement of wild type exon (blue) by GOF-carrying exon (cyan). (B) Mechanically activated currents and inactivation time constant (tau) in DRG sensory neurons from wild type (black) and constitutively homozygous GOF *Piezo2* mice (red). 87% WT and 70% GOF neurons were responsive to the stimulus. (C) Mechanically activated currents and inactivation time constant in Tdtomato+ neurons from *Pvalb*^*Cre*^/*Ai9* (wild type, black) and GOF *Piezo2; Pvalb*^*Cre*^*/Ai9* mice (red). (D) Bar graphs representing inactivation time constant (ms) and remaining current at the end of the stimulus (% of Imax value) in DRG sensory neurons. *p < 0.05, **p < 0.01, and ***p < 0.001 (Student’s t-test). Each data point represents a single DRG neuron.

We engineered mice that conditionally express a GOF *Piezo2* mutant to study the disease mechanism for DA5. One such human GOF mutation E2727del is in the C-terminus of PIEZO2 and it causes slower channel inactivation (5). We designed a knock-in strategy that allows Cre recombinase-dependent replacement of a wild type C-terminal exon of PIEZO2 with one that harbors the human-equivalent mouse GOF variant E2799del (Fig. 1A). We first generated constitutive GOF *Piezo2* mice (GOF^const.^) by breeding mice homozygous for the GOF allele into *Cmv*^Cre^ driver that expresses Cre recombinase ubiquitously (11). We used whole-cell patch clamp to record PIEZO2-dependent mechanically activated currents from dorsal root ganglion (DRG) neurons of homozygous GOF *Piezo2* mice and wild type littermates (Fig. 1B and Fig. S1A). We initially tested homozygous instead of heterozygous mice to avoid diluting the effects of the GOF allele by a wildtype allele. Although the majority of homozygotes died perinatally for unknown reasons, we were able to obtain DRGs from viable mice for recording. DRG neurons from homozygous GOF^const.^ mice showed a slower inactivation and a remaining current at the end of the stimulus (as a percentage of the peak current at the strongest stimulus applied) compared to wild type (Fig. 1B and Fig. S1B). As expected, there was no detectable difference in I_max_ and apparent threshold between wild type and GOF neurons (Fig. S1C). We also compared mechanically activated currents in DRG neurons of heterozygous mice to wild type to reflect the human condition. To enrich the recorded population for PIEZO2-dependent rapidly inactivating neurons, we crossed mice carrying the GOF *Piezo2* allele into *Pvalb*^*Cre*^*/Ai9*, which express Cre recombinase and the Ai9 tdTomato reporter protein in proprioceptive neurons (7, 12). We observed robust differences in the kinetics of mechanically activated currents between heterozygous GOF and wild type DRGs (Fig. 1C and D). These results validate that the engineered mice express a functional GOF PIEZO2 channel with similar kinetics of inactivation as human GOF mutations analyzed in heterologous system (5). They also demonstrate that heterozygosity of GOF PIEZO2 is sufficient to increase mechanosensitivity in sensory neurons.

## Overactive PIEZO2 causes limb defects in mice.

The constitutive heterozygous GOF mice had normal body weight at different ages (Fig S2A), suggesting that overactive PIEZO2 is unlikely to impact overall growth. In addition, food intake and metabolic rate of these transgenic mice were comparable to those of wild type littermates over a period of eight days (Fig S2B and C), suggesting normal eating behavior and metabolism.

**Figure 2.**
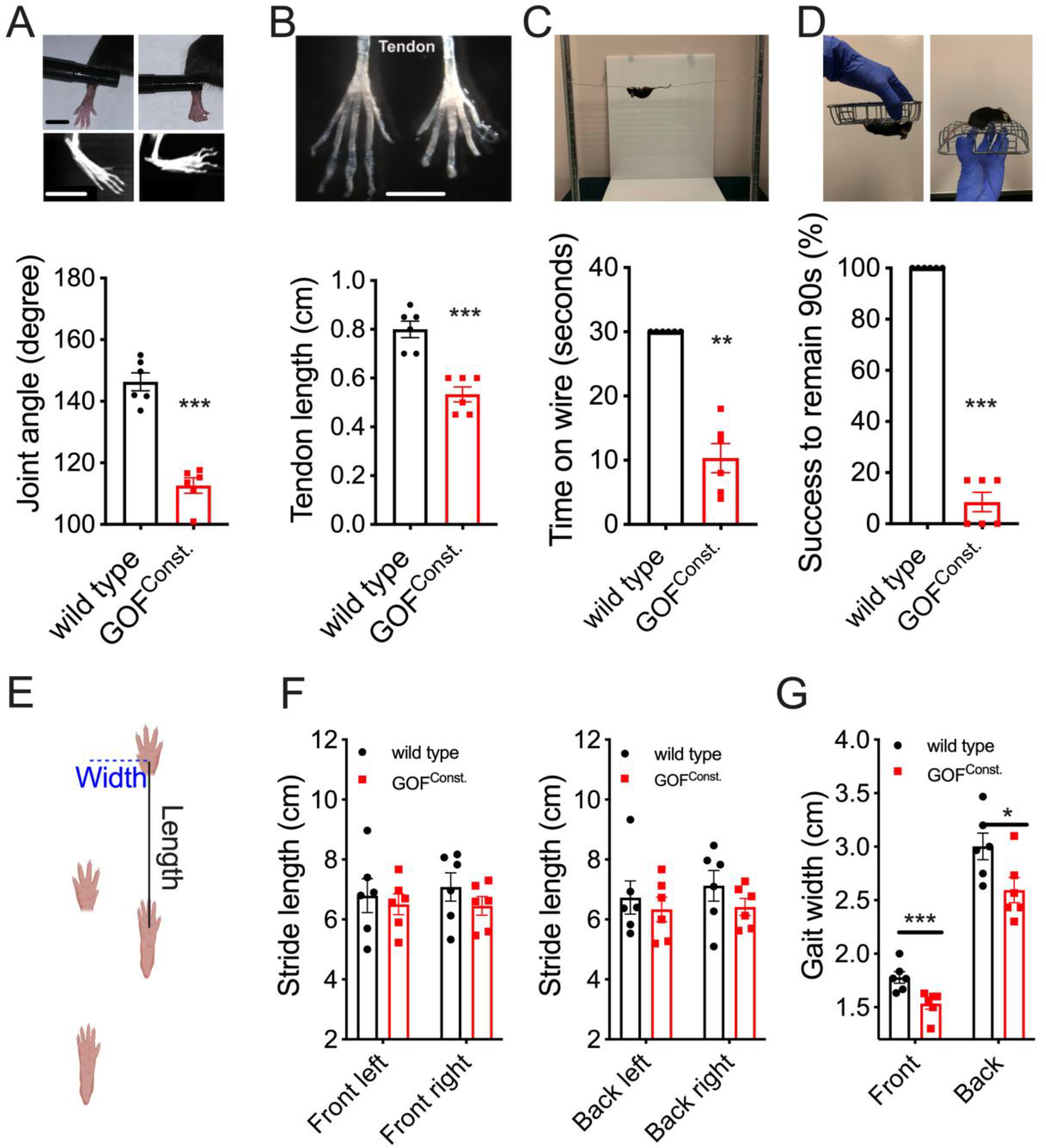
GOF *Piezo2* mice develop limb defects. (A) Joint morphology of live animals and CT scan (upper images) and phalange-metacarpal joint angles (lower graph) in the hindlimb. Left: wild type; Right: GOF mice. Scale bars: 0.5cm. (B) Intact tendon (upper) and tendon length (lower) from hindlimbs. Scale bar: 0.5cm. (C) Hanging wire test (upper) and Time (sec) that animals remained on the metal wire, with 30 sec as cutoff (lower). (D) Inverted screen test (upper) and quantification as percentage of animals successfully remaining on the rotating screen for 90 seconds (lower). (E) Gait assay: the stride length measures forelimb-hindlimb distance in the first (front) and second (back) stride. Gait width is the distance between forelimbs (front) or between hindlimbs (back) (F-G) Quantifications for E. *p < 0.05, **p < 0.01, and ***p < 0.001 (Student’s t-test). Each data point represents a single animal.

We observed that hindlimbs of the transgenic mice had contractures compared to wild type littermates (Fig. 2A). Direct visualization as well as Micro-computed tomography (CT) images showed that the angles between major hindlimb joints were smaller in GOF^const.^ mice than in wild type (Fig. 2A, upper panel). We measured the angle between the phalange and metacarpal, the two major bones of hindlimbs (it is challenging to quantitatively study forelimbs due to their small size) and found that phalange-metacarpal angles in hindlimbs of GOF^const.^ mice were significantly decreased compared to wild type mice (Fig. 2A, bar graph). DA5 patients have weak or absent tendon reflexes suggesting that defective tendon development might be associated with joint contractures (5). To evaluate tendon phenotypes, we dissected the intact tendons from hindlimbs (Fig. 2B), and we observed that the average length of tendons was significantly reduced in GOF^const.^ mice compared to wild type (Fig. 2B). It is therefore possible that the DA5-like contractures are caused by shortened tendons.

Anatomical deformities cause limited joint movements and affect motor functions in distal arthrogryposis patients. To test whether GOF PIEZO2 impacts limb functionality in mice, we performed two behavioral tests. First, we used a hanging-wire assay (13). The test is based on the latency of a mouse to fall off from a metal wire (Fig. 2C). We found that wild type mice were able to remain on the wire for at least 30 seconds, whereas GOF^const.^ mice fell off significantly earlier (Fig. 2C). Second, we performed the inverted screen test (14), which measures the time until a mouse falls off a square screen after it is turned upside down (Fig. 2D and method). All wild type mice were able to hold on for at least 90 seconds. In contrast, the average success rate for GOF^const.^ mice was less than 10% (Fig. 2D). Thus, these results suggest that limb functionality was compromised in GOF^const.^ mice, consistent with the anatomical defects.

We also measured the gait of GOF *Piezo2* mice (Fig 2E). The length of the stride during locomotion was similar to that of wild type (Fig 2F). In contrast, the width of their stride was decreased compared to wild type (Fig 2G). This is expected given the anatomical deficits, and is reminiscent of limited range of limb motion observed in DA5 human patients (5). Despite these defects, GOF *Piezo2* mice had normal daily activity patterns in an open field arena over eight days (Fig S2D), suggesting that overactive PIEZO2 is not completely debilitating.

### Somatosensory PIEZO2 overactivity causes limb defects.

**Figure 3.**
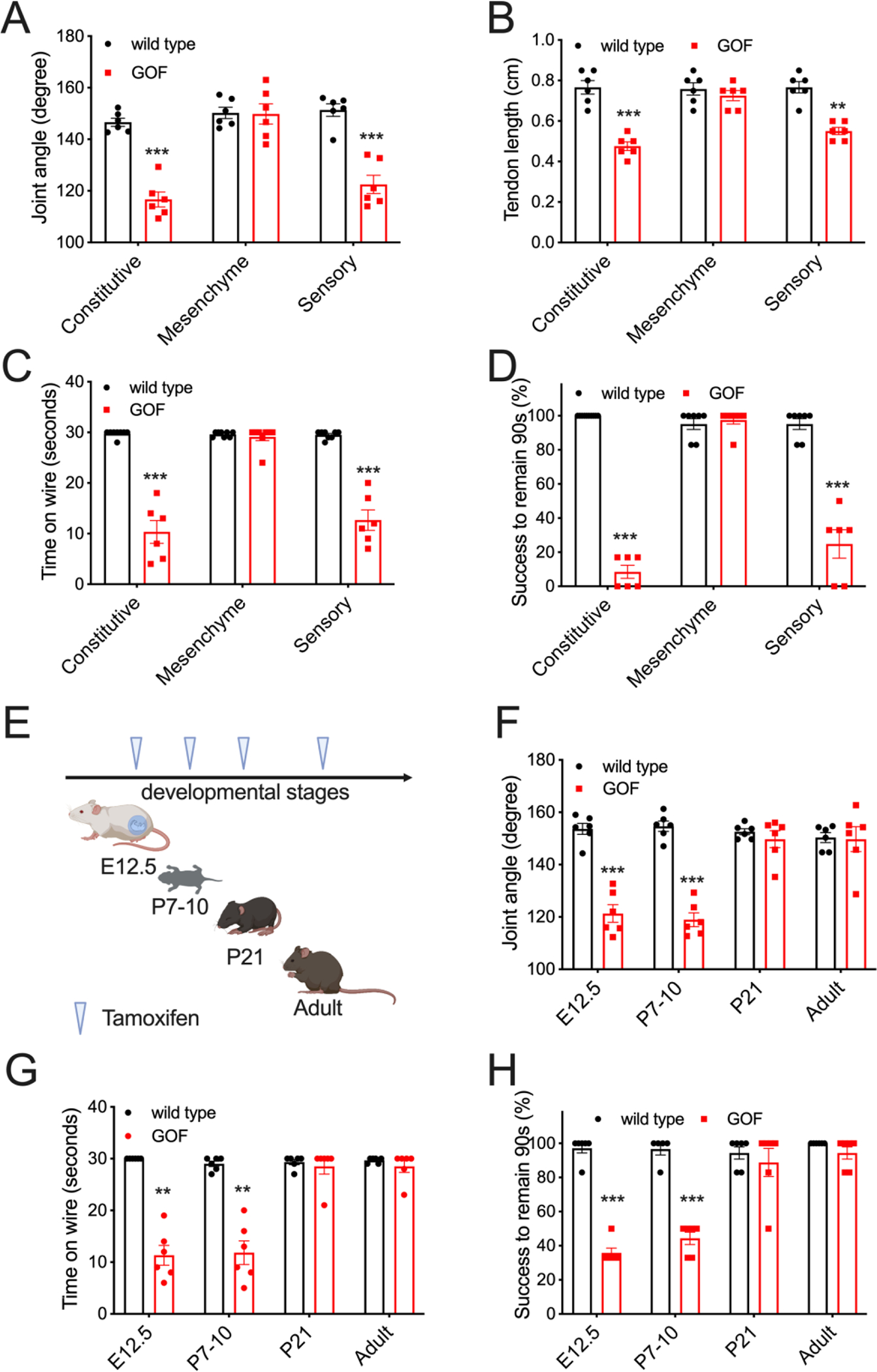
GOF PIEZO2 in sensory neurons causes limb defects. (A) Phalange-metacarpal joint angle of the hindlimbs in wild type and tissue specific GOF mice (black: wild type; red: GOF *Piezo2*). (B) Tendon length of hindlimbs. (C) Quantification of hanging wire test results. (D) Quantification of inverted screen test results. (E) Tamoxifen induction of GOF PIEZO2 in proprioceptive neurons by *Advillin*^*Cre-ERT2*^ at various developmental stages. (F) Phalange-metacarpal joint angle of the hindlimbs in E. (G) Quantification of hanging wire test results in E. (H) Quantification of inverted screen test in E. *p < 0.05, **p < 0.01, and ***p < 0.001 (One-way ANOVA followed by Tukey’s multiple comparison). Each data point represents a single animal.

To investigate which cell types are involved in the DA5 etiology, we expressed GOF *Piezo2* allele in various tissues. We induced GOF PIEZO2 expression in all mesenchymal cells including skeletal muscles, cartilages and tendons using *Prrx1*^Cre^ (15, 16). This Cre line induces robust recombinase activity in mouse limb mesenchyme starting from early embryogenesis. These GOF *Piezo2* mice had normal phalange-metacarpal angles and tendon lengths in their hindlimbs (Fig. 3A and B). Consistently, these mice performed comparably to wild type mice on behavioral assays (Fig. 3C and D). On the other hand, we found that expression of GOF PIEZO2 in proprioceptive sensory neurons (via *Pvalb*^*Cre*^ knock-in mice) that mainly innervate muscle spindles and tendon organs (7, 17) was sufficient to cause joint contractures, shortened tendons, and poor performance on behavioral assays (Fig. 3A–D). These data suggest that overactive PIEZO2 in sensory neurons, but not in mesenchyme, is sufficient to induce DA5-like phenotypes *in vivo.*

Since DA is a developmental disorder presumably originating during fetal stages (2), we used our mouse model to determine the timing of PIEZO2 activity in sensory neurons that affects musculoskeletal development. We used an inducible sensory neuron-specific GOF *Piezo2* mouse by crossing the GOF allele into *Advillin*^Cre-ERT2^ driver mice (18). Advillin is expressed in most DRG neurons, but not central nervous system or non-neuronal cell types, starting in early embryogenesis (19, 20). We induced GOF PIEZO2 expression in sensory neurons at various developmental stages by tamoxifen injection (Fig. 3E). When induced at embryonic day 12.5 or at postnatal day 7–10 (P7–10), these mice showed significantly decreased phalange-metacarpal joint angles (Fig. 3F), suggesting that the phenotype is mainly dependent on expression of PIEZO2 sometime after P7. However, induction of GOF PIEZO2 expression in sensory neurons after P21 or during adulthood (> 1month old) did not cause joint defects in mice (Fig. 3F). Consistent with anatomical phenotypes, mice expressing GOF PIEZO2 in sensory neurons during embryonic (E12.5) and early postnatal stage (P7–10), but not after early adulthood (>P21), performed poorly on behavioral tests (Fig. 3G and H). These results confirm our earlier findings using *Pvalb*^Cre^ that overactive PIEZO2 in sensory neurons causes DA5-like defects (Fig. 3A–D). In addition, they suggest a critical postnatal developmental period between days 7 and 21 during which excessive mechanotransduction in somatosensory neurons causes limb malformation.

To ensure that the anatomical deficits are not an indirect consequence of compromised proprioceptive neuronal development, we examined proprioceptive nerve endings of muscle spindles and Golgi tendon organs (Fig. S3). We did not observe overt anomalies in these proprioceptive endings (Fig. S3A). In addition, we quantified the size of proprioceptors innervating muscle spindles and observed no difference between GOF *Piezo2* and wild type mice (Fig. S3B). Further, we did not find significant changes in extrafusal and intrafusal muscle fiber diameters in GOF *Piezo2* mice (Fig. S3C), suggesting that overactive PIEZO2 in sensory neurons does not appear to have strong effects on muscle development.

## Overactive PIEZO2 causes defects via efferent pathways.

**Figure 4.**
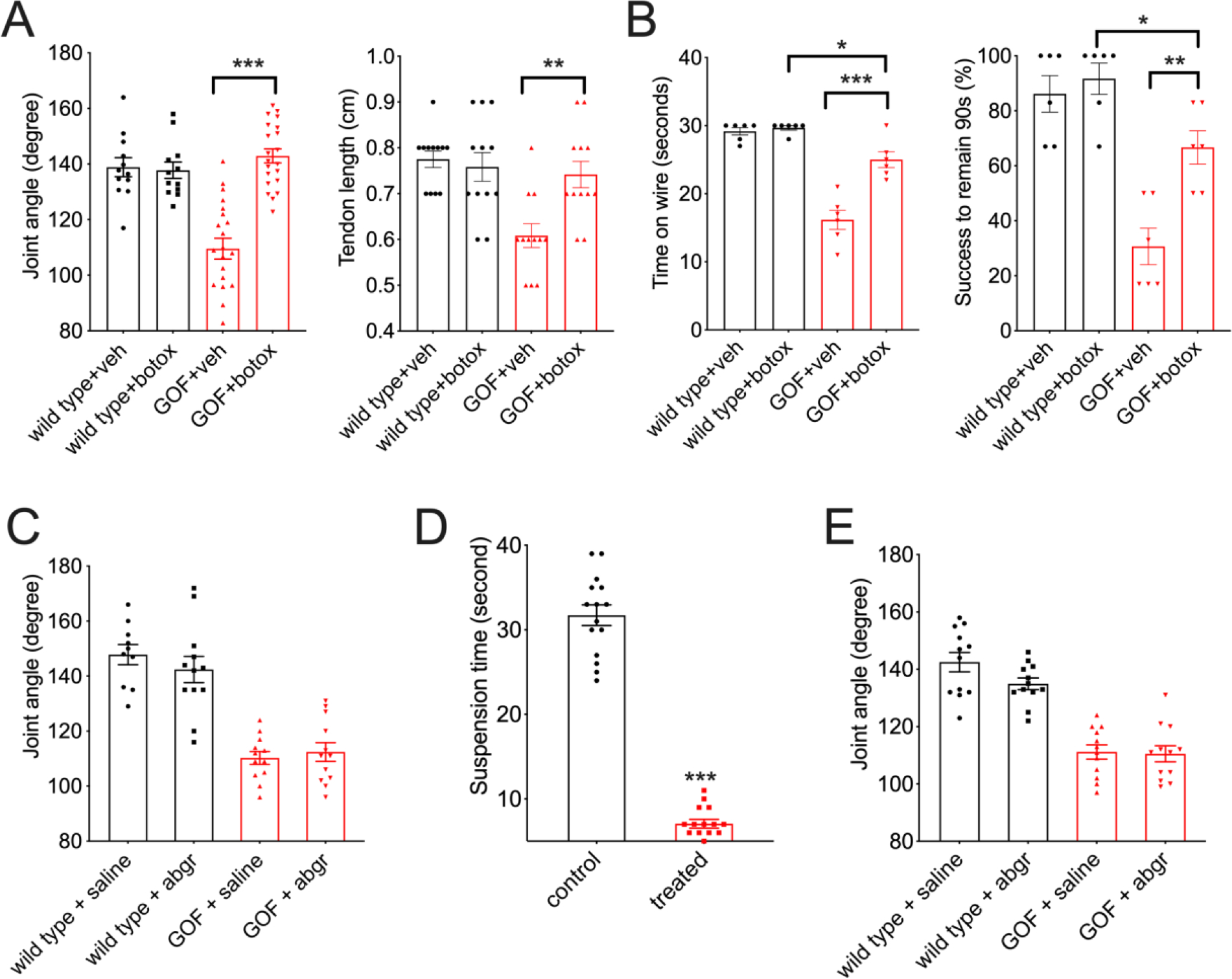
Pharmacological effects on limb phenotypes in GOF mice. (A) Phalange-metacarpal joint angle and tendon length of the hindlimbs in young adult wild type and GOF *Piezo2*; *Pvalb*^*Cre*^ mice that received Botulinum toxin (Botox) at P7–10. (B) Quantification of behavioral tests in mice treated with Botox. (C) Phalange-metacarpal joint angle of the hindlimbs in young adult mice receiving a single dose of alpha-Bungarotoxin (abgr) at P7–10. (D) Quantification for motor defects (in a modified hanging assay) in young adult mice receiving 5-day alpha-Bungarotoxin (abgr) treatment at P7–10. (E) Phalange-metacarpal joint angle of the hindlimbs in young adult mice receiving daily alpha-Bungarotoxin (abgr) treatment from P5–10 in D.

We also used a pharmacological approach to further test whether anatomical defects of GOF *Piezo2* mice are attributed to hyperactivation of sensory neurons. We performed intra-muscular injection of Botulinum toxin (Botox) into the hindlimb of mice at P7–10 (Fig. 4A), at the beginning of the critical period. The contralateral hindlimb received vehicle injection as an internal control. Botox blocks neural transmission from both sensory and motor neurons by inhibiting exocytosis (21). Four weeks after a single dose of injection, we observed that Botox did not affect joint morphology and tendon length in wild-type mice (Fig. 4A). Meanwhile, it rescued joint and tendon defects in the hindlimb that received the treatment in GOF *Piezo2* mice compared to hindlimb injected with vehicle control (Fig. 4A). Further, if administered on both sides of hindlimbs, Botox improved performances on the behavioral assays for GOF *Piezo2* mice (Fig. 4B). However, it failed to completely restore the behavioral outcomes, probably due to deficits in forelimbs which did not receive Botox treatment. This result suggests that GOF PIEZO2-dependent neuronal activity is responsible for joint defects.

Mechanical activation of proprioceptive nerve endings has two consequences: 1. it causes local Ca^2+^-dependent signaling within the peripheral proprioceptive endings culminating in exocytosis (22), and 2. it induces an afferent signal by initiating firing of proprioceptive neurons, which activates downstream motor neurons innervating the same peripheral muscle to counteract the stretch (23). Botulinum toxin would block both these processes. To test whether the results we observed using Botox resulted from blocking motor neuron output, we injected ɑ-Bungarotoxin, which blocks neural transmission from motor neurons to muscles (24), into hindlimbs of P7–10 GOF *Piezo2* mice. This treatment did not alleviate anatomical defects in these mice (Fig. 4C). It is possible that a single dose of ɑ-Bungarotoxin is insufficient to inhibit motor transmission throughout the critical developmental period because it has shorter pharmacological window than Botox (25). To assess this possibility, we injected ɑ-Bungarotoxin into the hindlimbs for five consecutive days. This treatment was sufficient to severely impact motor neuron function, as it compromised motor behavior (Fig. 4D), but it still failed to rescue anatomical defects in GOF *Piezo2* mice (Fig. 4E). This implies that motor activity does not play a major role in DA5 like deficits. Together, our pharmacological experiments suggest that GOF PIEZO2 causes musculoskeletal abnormalities via ectopic efferent activity in afferent peripheral mechanosensory neuronal endings, likely involving increased levels of exocytosis.

We also tested whether GOF PIEZO2 allele caused increased proprioceptive firing (afferent function) during development. To measure muscle spindle firing, we evaluated proprioceptor mechanotransduction *ex-vivo*. In this assay, we mechanically stretched the extensor digitorum longus (EDL) muscle and recorded from the innervating proprioceptors (muscle spindles) to assess stretch-induced neuronal activity (Fig. S4A) (7, 26). We found that muscle spindle afferent activity in response to stretch is not significantly different between wild type and GOF *Piezo2* mice at P7 postnatal stage (Fig. S4B–C). Therefore, we observe increased PIEZO2 current in cell culture assays, but no increased neuronal firing *ex vivo*. It is possible that somatosensory neurons have compensatory mechanisms for controlling overactive transduction responses. Thus, these recordings are consistent with the idea that local efferent action of afferent proprioceptive neurons caused by overactive PIEZO2 is responsible for the developmental deficits.

### EPA diet alleviates defects in GOF mice.

Our data suggests that manipulations that would decrease overall PIEZO2 activity during a critical development time could potentially rescue the DA5-like phenotype. The ω-3 polyunsaturated fatty acid (PUFA) eicosapentaenoic acid (EPA), a membrane lipid component, significantly decreases the inactivation time of wild type and GOF PIEZO1 channels, a close relative of PIEZO2 (27, 28). Because EPA is commonly found in fish and widely used as a dietary supplement, we tested whether a diet enriched in ω-3 PUFAs has therapeutic potential.

**Figure 5.**
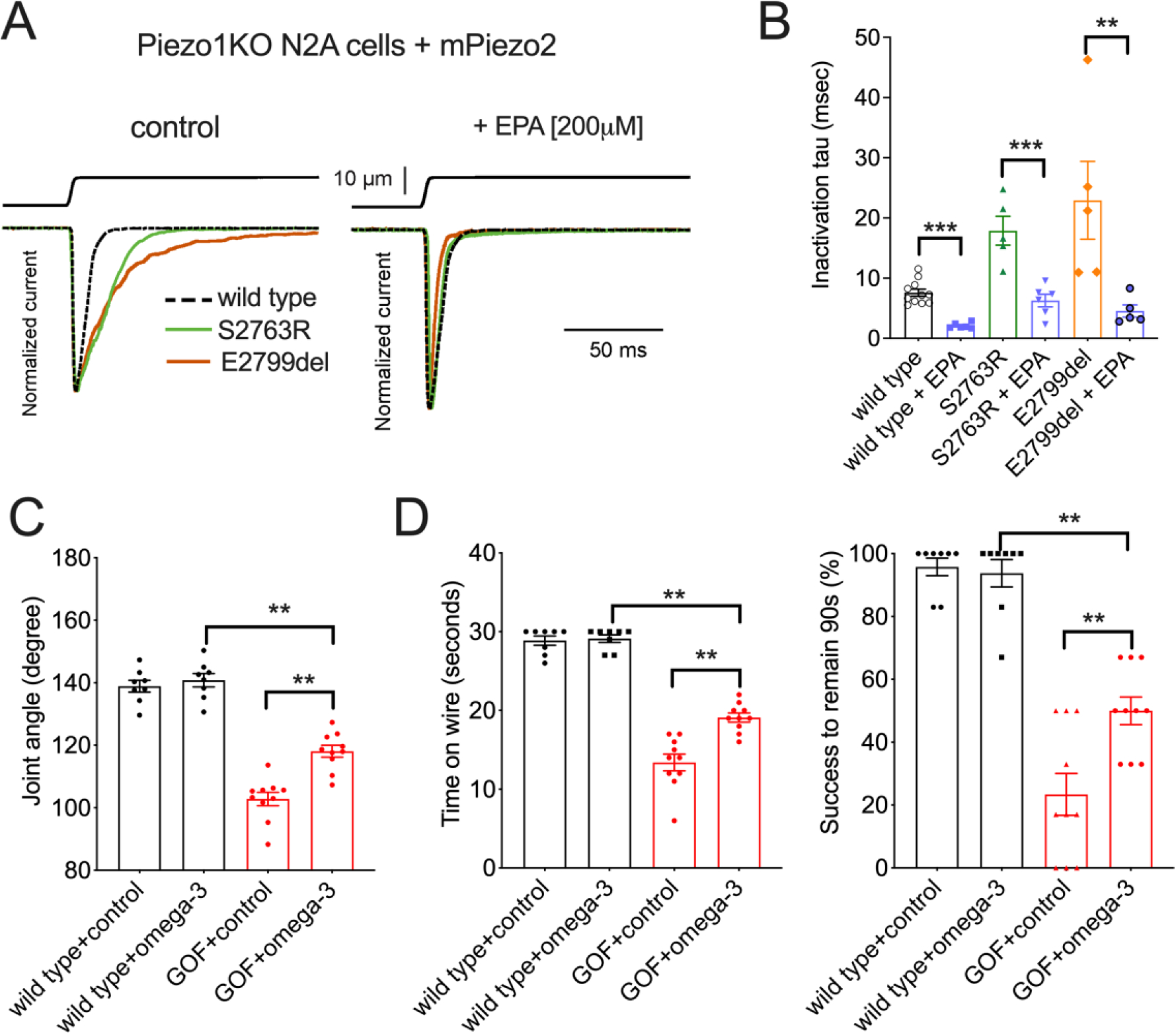
EPA diet rescues joint defects in GOF mice. (A) Mechanically activated currents in N2A*Piezo1*−/− cells heterologously expressing mouse PIEZO2 wild type (stippled) and GOF mutants S2763R (green) and E2799del (brown). (B) Quantification of the inactivation time constant results in A. (C) Phalange-metacarpal joint angle of the hindlimbs in young adult wild type and GOF *Piezo2*; *Pvalb*^*Cre*^ mice that received EPA-enriched diet. Control: sunflower-oil-based food, which has a similar level of fat content and energy. (D) Quantification of hanging wire and inverted screen test results in C. *p < 0.05, **p < 0.01, and ***p < 0.001 (Two-way ANOVA followed by Tukey honestly significance test).

We first showed that overnight incubation with EPA (200 μM) reduced wild type as well as GOF mutant PIEZO2 inactivation time constant in a heterologous expression system (Fig. 5A and B). This suggests that EPA has the potential to modulate disease causing mutations in PIEZO2. We then fed female mice a diet enriched in EPA during pregnancy and nursing. The sensory neurons of their offspring were later assessed for PIEZO2-dependent mechanosensitivity (Fig. S5A). We found that EPA-enriched diet intake significantly reduced inactivation time constant of mechanically stimulated currents in sensory neurons but had no effect on the current intensity and apparent threshold (Fig. S5A). Next, we determined the effect of this diet on the anatomical defects in GOF *Piezo2* mice. Liquid chromatography-mass spectrometry (LC-MS) showed that the feeding protocol successfully incorporated EPA into the plasma membrane of sensory neurons that carry the GOF *Piezo2* mutation (Fig. S5B). Finally, we observed that dietary EPA partially prevented joint malformations (Fig. 5C) and improved the performances on the hanging wire and inverted screen tests (Fig. 5D) in GOF *Piezo2* mice.

Our study has identified a critical postnatal period during development when relatively small excess PIEZO2 activity in somatosensory neurons can cause tendon deficits and severe joint contractures. Previous work has shown that loss of PIEZO2 in proprioceptive neurons causes skeletal abnormalities manifested as spine malalignment in mice (15), indicating a requirement of proprioceptive feedback for proper vertebral development. We hypothesize that overactive PIEZO2 causes anatomical abnormalities in joints via increased exocytosis from sensory neuron endings without involving motor circuitry. Indeed, it is known that sensory neurons secrete a variety of factors in response to neuronal stimulation, including glutamate release from proprioceptive endings (22). While the role of secreted factors such as calcitonin-gene-related peptide (CGRP) from nociceptors in mediating pain and inflammation is well documented (29), relatively little is known about the importance and identity of such efferent signaling molecules in other sensory neurons, including proprioceptors.

We also present proof-of-concept that Botox injection or dietary treatment can counteract the effect of overactive PIEZO2 function to evade DA-like phenotypes in mice when applied during a developmental critical period. These approaches might have clinical applications. Beyond this, our findings call attention to the importance of considering sensory mechanotransduction when diagnosing and treating other musculoskeletal disorders.

## Supplementary Material

1

## Data Availability

GOF *Piezo2* mice (C57BL/6NTac-*Fam38b*^*tm3287*^*(ED2799D)Arte* are available from Ardem Patapoutian upon request or directly from Taconic Biosciences. All data are available in the main text or the supplementary materials.
