# Supplementary material for: Excessive Mechanotransduction in Sensory Neurons Causes Joint Contractures
Source: Science. Author manuscript; Available in PMC 2023 May 6. (PMC10163824; doi:10.1126/science.add3598)
Supplement: 1 [file NIHMS1893124-supplement-1.pdf]

Materials and Methods

Animals

All animal procedures were approved by the Institutional Animal Care and Use Committees of The Scripps Research Institute (California), San José State University, and the University of Tennessee Health Science Center. Mice carrying the conditional gain-of-function (GOF) *Piezo2* allele were generated and maintained on C57BL/6 background. All animals were backcrossed at least 10 generations into C57BL/6. Constitutive GOF *Piezo2* ($GOF^{const.}$) mice were generated by breeding mice with conditional GOF allele to Cmv^{Cre} driver (The Jackson Laboratory, stock #006054). Tissue specific GOF *Piezo2* mice were generated by breeding conditional GOF mice into MCK^{Cre} (The Jackson Laboratory, stock#006475), $Prrx1^{Cre}$ (The Jackson Laboratory, stock#005584), $Pvalb^{Cre}$ (The Jackson Laboratory, stock#017320) and $Advillin^{Cre\text{-}ERT2}$ (The Jackson Laboratory, stock#026516) driver mice, respectively. Reporter mouse Ai9 was from The Jackson Laboratory, stock#007909. Mice were housed in a temperature-controlled (22-24°C) room that maintains a 12hr light/dark cycle. All tissue harvests and behavioral assays were performed on mice between 1.5 to 6 months old. Pharmacological treatments were done on mice at different ages as specified (see below for details).

Electrophysiology and mechanical stimulation

DRG neurons.

Mechanically activated currents from DRG neurons 1-3 days after culturing were recorded in whole cell patch clamp mode using a MultiClamp700A amplifier and DigiData1550 (Molecular Devices) and stored directly and digitized online using pClamp

software (version 10.7). Currents were sampled at 20 kHz and filtered at 2 kHz. Recording electrodes had a resistance of 3 to 7 Megohms when filled with gluconate-based low-chloride intracellular solution: 125 mM K-gluconate, 7 mM KCl, 1 mM $CaCl_2$, 1 mM $MgCl_2$, 10 mM HEPES (pH with KOH), 1 mM tetra-K BAPTA [1,2-bis(2-aminophenoxy)ethane-N,N,N′,N′-tetraacetic acid], 4 mM Mg-ATP (adenosine triphosphate), and 0.5 mM Na-GTP (guanosine triphosphate). Extracellular bath solutions used: 133mM NaCl, 3mM KCl, 2.5mM $CaCl_2$, 1mM $MgCl_2$, 10mM HEPES (pH7.4), and 10mM glucose.

Mechanical stimulation was achieved using a fire-polished glass pipette (tip diameter, 3 to 4 µm) positioned at an angle of 80° relative to the cell being recorded. Displacement of the probe toward the cell was driven by Clampex-controlled piezoelectric crystal microstage (E625 LVPZT Controller/Amplifier; Physik Instrumente). The probe had a velocity of 1 µm msec−1 during the ramp phase of the command for forward movement, and the stimulus was applied for a duration of 125 msec. For each cell, a series of mechanical steps in 1-µm increments was applied every 10 sec.

Heterologous expression in N2A$^{Piezo1-/-}$ cells.

For whole-cell recordings of mechano-activated currents in Piezo1-/- N2A cells and mouse pups DRG neurons in EPA -related experiments, the bath solution contained 140 mM NaCl, 6 mM KCl, 2 mM CaCl2, 1 mM MgCl2, 10 mM glucose, and 10 mM HEPES (pH 7.4), while the pipette solution contained 140 mM CsCl, 5 mM EGTA, 1 mM CaCl2, 1 mM MgCl2, and 10 mM HEPES (pH 7.2). Pipettes were made of borosilicate glass (Sutter Instruments) and fire-polished to a resistance between 3 and 5 MΩ before use. Mechanical stimulation was performed using the voltage-clamp (constant -60 mV)

configuration. Recordings were sampled at 100 kHz and low pass filtered at 10 kHz using a MultiClamp 700 B amplifier and Clampex (Molecular Devices, LLC). Leak currents before mechanical stimulations were subtracted offline from the current traces, and data were digitally filtered at 2 kHz with ClampFit (Molecular Devices, LLC). Recordings with leak currents > 200 pA, with access resistance >10 MΩ, and cells with giga-seals that did not withstand at least five consecutive steps of mechanical stimulation were excluded from analyses.

Other buffers

CsCl-based intracellular solution: 133 mM CsCl, 1 mM CaCl2, 1 mM MgCl2, 10 mM HEPES (pH with CsOH), 5 mM EGTA, 4 mM Mg-ATP (adenosine triphosphate), and 0.5 Na-GTP (guanosine triphosphate). Extracellular bath solution was composed of 133 mM NaCl, 3 mM KCl, 2.5 mM CaCl2, 1 mM MgCl2, 10 mM HEPES (pH 7.3 with NaOH), and 10 mM glucose.

N2A cell transfections

Neuro2A cells were co-transfected with 250-500 ng*mL^{-1} of mouse Piezo2 cDNA (wild type and S2691R and E2727del GOF mutants) and 50 ng/mL GFP-pMO, using Lipofectamine 2000 (Thermo Fisher Scientific) according to the manufacturer's instructions. Fatty acids were supplemented 18-24 hours before recording.

Liquid chromatography-mass spectrometry

DRG tissues were frozen in liquid nitrogen immediately after dissection from GOF *Piezo2* mice fed with standard, sunflower, or EPA-enriched diets. Total and free fatty acids were extracted and quantified at the Lipidomics Core Facility at Wayne State

University. Membrane (i.e., esterified) fatty acids were determined by subtracting free from total fatty acids and normalized by protein content

Anatomical analyses

Wild type and GOF mice littermates between 1.5 to 6 months old were euthanized by isoflurane. Distal part (feet) of hindlimbs from the mice was severed, with skins and calcaneus (heel bone) intact, followed by fixation in 4% PFA for one week at 4°C. The angle between phalange and metacarpal was measured for the middle three digits. The data was shown as the average for each mouse.

For tendon analysis, the intact flexor tendon was detached and dissected out from the bones and muscles of mouse hindlimb. The tendon length was measured as the distance from the tip of third (longest) flexor tendon to the attachment site to the flexor digitorum profundus.

Immunohistochemistry

Adult mice were perfused with phosphate-buffered saline (PBS) and 4% paraformaldehyde (PFA) followed by isolation of the skeletal muscle gastrocnemius and soleus muscles from the hindlimbs. Whole muscles were post-fixed in 4% PFA overnight in a rotating incubator in 4°C. Muscles were washed in 30mL PBS for 2h followed by 30 mL PBS with 0.3% TritonX-100 (PBST) for 6h. Whole muscles were blocked in 1% bovine serum albumin (BSA, wt/vol), 5% normal goat serum (NGS, vol/vol), and 0.3% PBST for 1h. Primary antibodies were incubated in 1% BSA, 5% NGS, and 0.3% PBST for 3 days at 4°C on rotating incubator (NFH, 1:500, Abcam, ab4680; Tubulin β-III, 1:200, Biolegend, 801201). Whole muscles were washed in 0.3% PBST for 5 hours then incubated in secondary antibodies for 48h at 4°C on rotating

incubator (AlexaFluor 568, 1:200, Invitrogen, A11041; AlexaFluor 647, 1:200, Invitrogen, A21236). Whole muscles were dehydrated in a series of 25%, 50%, and 75% MeOH in PBS for one hour each on shaking incubator at room temperature, protected from light. Whole muscles were dehydrated in 100% MeOH overnight on shaking incubator at room temperature. Muscles were incubated in 1-part benzyl alcohol to 2-part benzyl benzoate for 1 hour then whole mounted for imaging. To quantify the size of proprioceptors innervating muscle spindle, we used ImageJ to calculate all areas covered by fluorescence from proprioceptor immunostaining. Intrafusal fibers were identified as those wrapped by vGlut1+ and NF+ proprioceptive nerves while other muscles were considered as extrafusal fibers. The width of the muscle fiber mid-point was measured as fiber diameters in the whole-mount images.

Behavioral tests

Hanging wire assay.

A 60cm long and 2mm thick metal wire was secured to two vertical poles. The wire was tightly attached to the poles to avoid displacement and vibration while the mice were being assayed on the wire. Also, the wire was about 40cm above a layer of soft bedding. The suspension time for each mouse was measured in three trials with one minute recovery period between trials, and the average was calculated. The time until the animal completely fell off from its grasp was recorded. Because all wild type mice were able to remain on the wire for at least 30 seconds while GOF mice had significantly shorter suspension time, 30-second was used as a cutoff.

Inverted screen test.

Mice were placed individually on the top of a metal square (around 8cm x 8cm) grid screen. The screen was then rotated 180° and the mice were on the bottom of the screen. The suspension time on the screen (either remaining on the bottom or climbing up to the top side) was recorded for each mouse in six trials, with one minute recovery period between trials. The percentage of successful trials with at least 90-second suspension time was calculated for each mouse.

Pharmacological experiments

Botulinum toxin A (Botox, 100 units) was purchased from Allergan (Irvine, CA) and diluted by water to a working solution of 5U/ml. ɑ- Bungarotoxin (Sigma-Aldrich, St. Louis, MO) was diluted by PBS to working solution of 100ug/ml. Wild type and GOF Piezo2; Pvalb-Cre mice at P7-10 were briefly immobilized on ice immediately before drug injection. Botox was injected into two sites of the hindlimb: one dose (0.5U kg^{-1}) via intramuscular injection into distal part of gastrocnemius and another dose (0.5U kg^{-1}) via intraplantar injection into the paws. Similarly, ɑ- Bungarotoxin (10ug kg^{-1}) was injected into these two sites of the hindlimb. For each animal, one side of hindlimb received drug injection while the contralateral side was injected with vehicle as an internal control. For behavioral experiments after Botox treatment, mice received vehicle or drug on both sides of hindlimbs at P7-10.

Muscle spindle afferent recordings

The extensor digitorum longus (EDL) muscle and deep peroneal branch of the sciatic nerve were dissected from P7-11 mice of both sexes, similarly to previous work (26). The tendons were sutured to a post and a lever arm connected to a dual force, length controller and transducer (300C-LR, Aurora Scientific). Resting length (Lo) of the EDL

muscle was measured before removal when both knee and ankle joints were 90º. A suction electrode (tip diameter 25–75 μm) connected to an extracellular amplifier with head stage (Model 1800, A-M Systems) was used to detect sensory activity. The nerve was sampled to find stretch sensitive sensory activity and a series of 9 ramp and hold stretches was given (3 stretches each to Lo plus 2.5%, 5% and 7.5% Lo; ramp speed 40% Lo s^{-1}; stretches held 4 s with 1 min in between each stretch). Sensory response to 60 twitch contractions at 1-Hz frequency was then sampled to identify a characteristic pause in muscle spindle afferent firing to confirm proprioceptor identity.

Tamoxifen injection

Tamoxifen was administered as previously published (6). Briefly, 150mg of tamoxifen (Sigma) was dissolved in 10ml of 100% corn oil (at 50ºC) freshly daily before use. Dissolved tamoxifen solution was injected intraperitoneally (at room temperature) into wild type and GOF *Piezo2*; *Advillin*$^{Cre\text{-}ERT2}$ mice (littermates) (see main text) at 150mg kg^{-1} for five consecutive days. Each mouse was weighted before injection to normalize for differences in body weight. Anatomical analyses and behavioral assays were performed between 4 to 6 weeks after tamoxifen injection.

Diet intervention

Breeding mice were fed with standard, menhaden (high EPA content, Ref), or sunflower (a control diet with similar caloric content) oil-enriched diets. For electrophysiology experiments, pregnant females, when reaching late pregnancy (2-3 days before birth), were relocated to a new cage and fed with regular rodent diet until their pups were 9 days old. The reason for this relocation was to reduce cannibalism observed when newborns and parents were in the same EPA-enriched diet feeding cage. DRGs from

P9 pups were dissected, cultured, and for mechanically activated (MA) currents. For analyses on mouse anatomy and behavior, parenting mice and newborn pups were housed in the same EPA-enriched diet feeding cage with a different bedding material. Cannibalism was reduced by this change and relocation was not necessary (i.e. these animals were fed EPA-enriched diet throughout their life). Anatomical and behavioral assays were performed when pups were 5 weeks old.

Statistical analysis

Statistical analyses were performed and significance was computed using GraphPad Prism 6. Student's t tests were used when comparison was made between two groups. Unpaired *t* test with Welch correction was used when comparing three groups. One-way and two-way ANOVA with multiple comparison tests were performed when comparing two groups, at multiple time or conditions. When specific tests have been made, it is stated in the figure legend. P values ($\alpha = 0.05$, two sided) are indicated in figure legends.

Figure S1

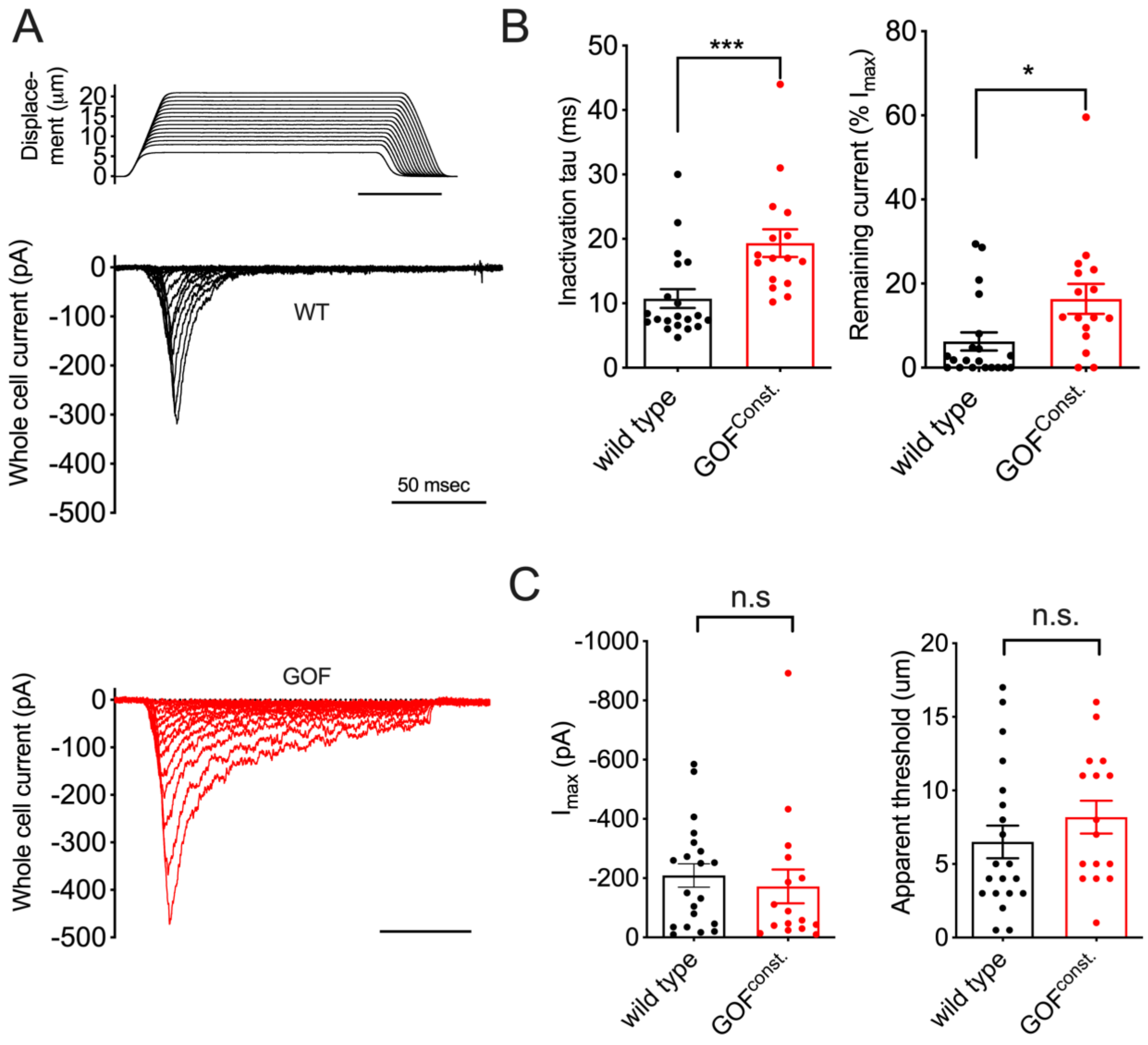

Fig. S1. (A) Representative traces of mechanically activated (MA) currents (whole -cell patch clamp recording) for wild type (WT, black) and homozygous constitutive GOF *Piezo2* DRG ($GOF^{const.}$, red) sensory neurons. (B) Quantification for I_{max} and apparent threshold of wild type (WT) and homozygous $GOF^{const.}$ DRG neurons. (C) Inactivation time constant (tau) and steady state current of MA currents from wild type (WT) and heterozygous GOF *Piezo2*; *Pvalb*^{Cre} ($GOF^{sensory}$) DRG neurons.

Figure S2

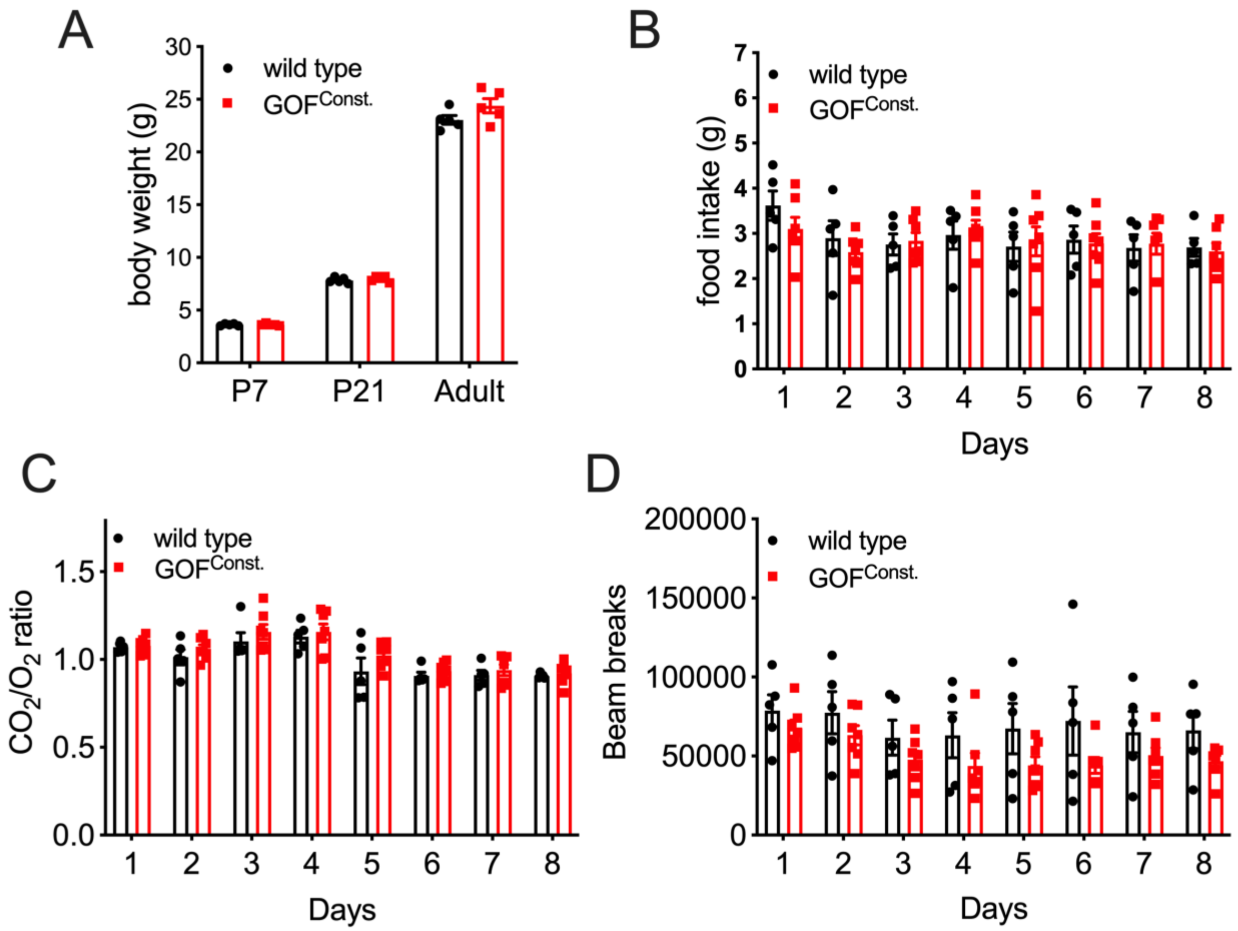

Fig. S2. (A) Body weights (gram) at different developmental stages. (B) Food intake (gram) per day for adult mice. (C) CO_2/O_2 ratio (by volume) for adult mice. (D) Open field test for adult mice. Animal activity is quantified by the number of laser beam breaks.

Figure S3

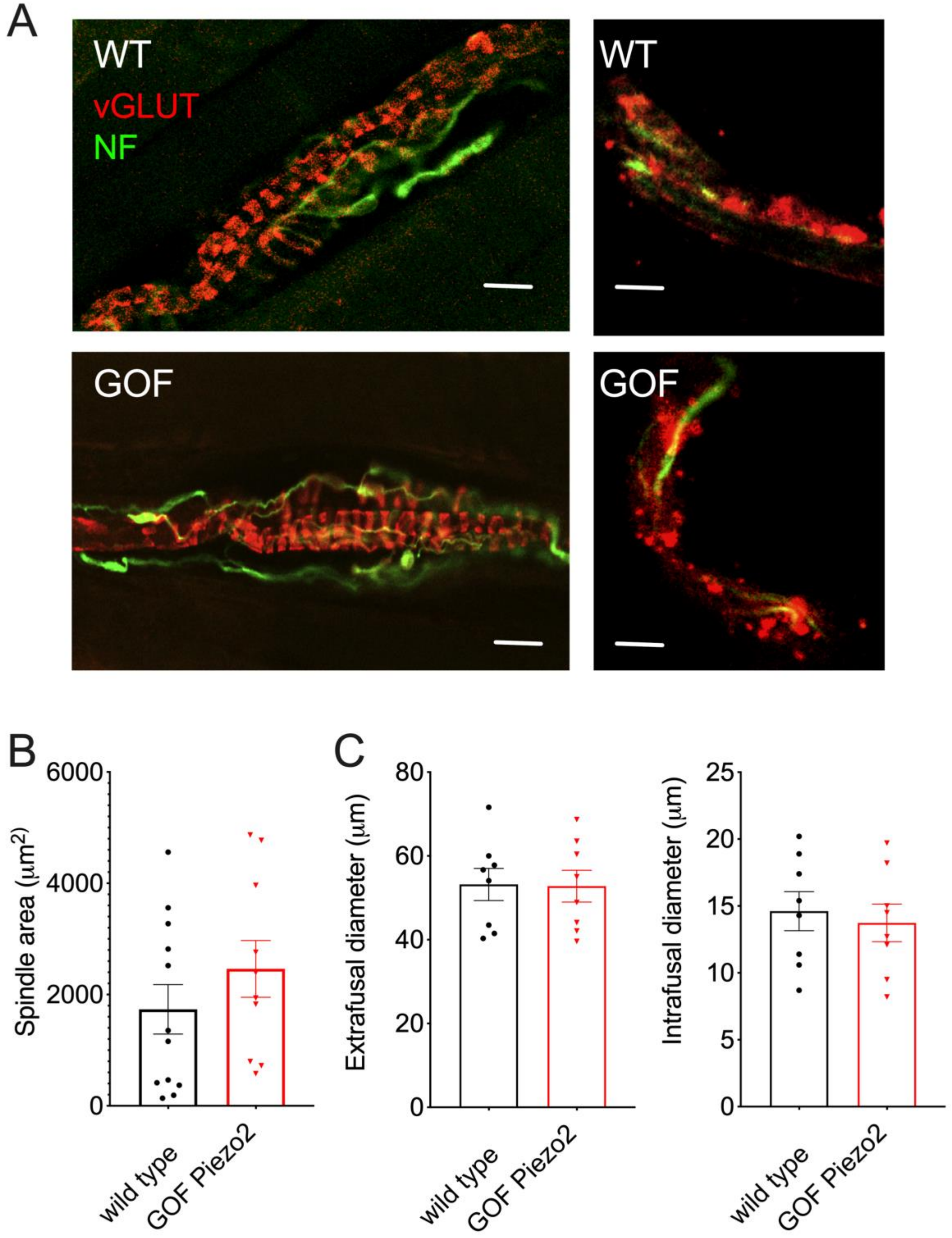

A
WT
vGLUT
NF
WT
GOF
GOF
B
6000
4000
2000
0
Spindle area (μm²)
wild type
GOF Piezo2
C
80
60
40
20
0
Extrafusal diameter (μm)
wild type
GOF Piezo2
25
20
15
10
5
0
Intrafusal diameter (μm)
wild type
GOF Piezo2

Fig. S3. (A) Morphology of proprioceptors in the skeletal muscles (left) and Golgi tendon organ (right) in wild type (WT) and GOF *Piezo2*; *Pvalb*^{Cre} mice. vGLUT1 (red) and neurofilament heavy chain (NF; green) immunohistochemistry showing proprioceptors. Representative images from three pairs of wild type and GOF mice. Scale bar: 20 μm. (B) Muscle spindle proprioceptor size quantified as areas of vGLUT1 staining. (C) Muscle fiber diameter in adult mice.

Figure S4

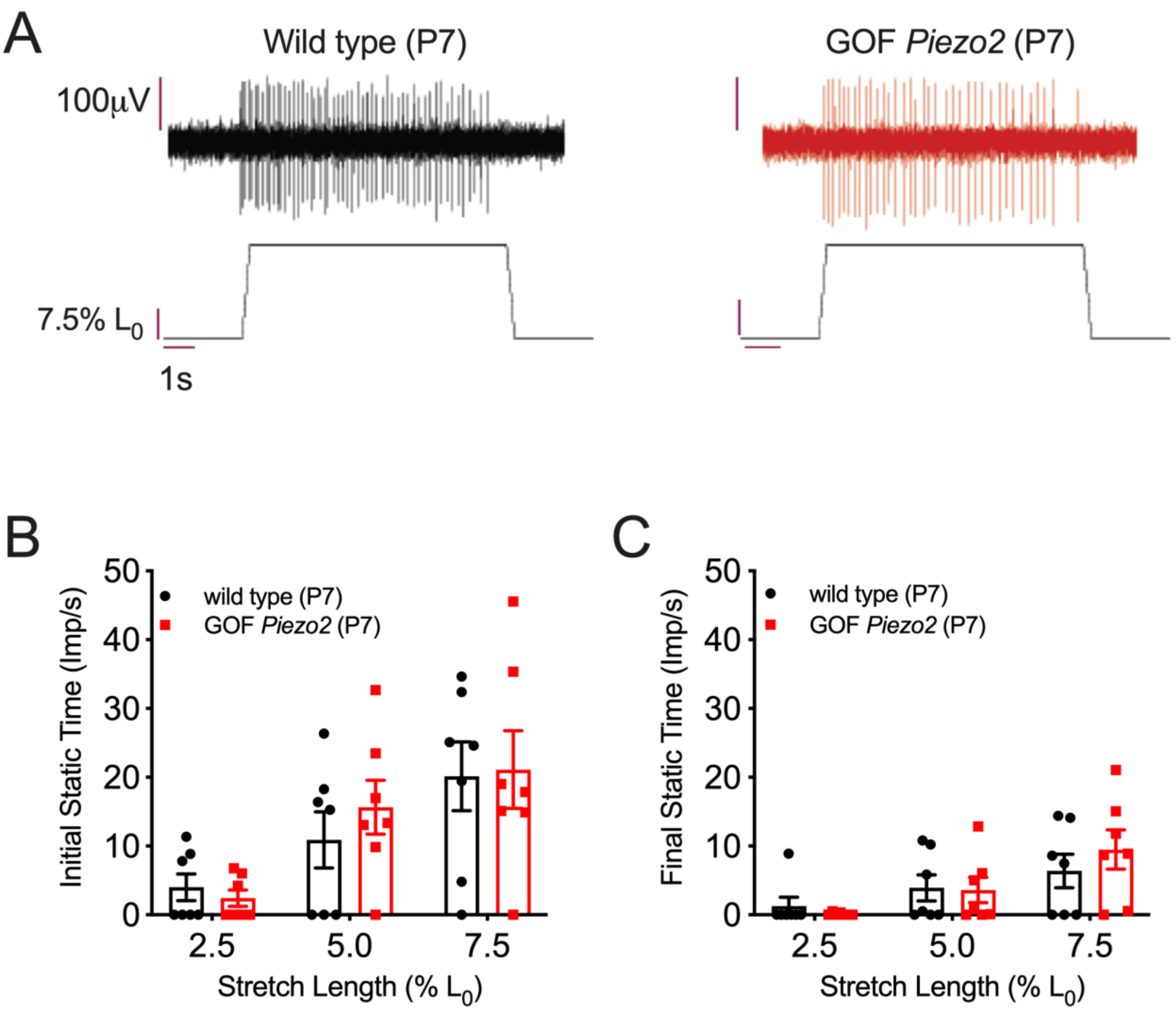

Fig. S4. (A) Representative response to stretch from wild type (left) and GOF *Piezo2* (right) muscles (at postnatal day 7). Firing frequency from identified muscle spindle afferents during the beginning. (initial static time or 0.5s into stretch). (B) and end of stretch (Final static time or 3.5s into stretch) (C) at different stretch lengths (% of muscle optimal length, or length where the maximum force of twitch contraction is generated).

Figure S5

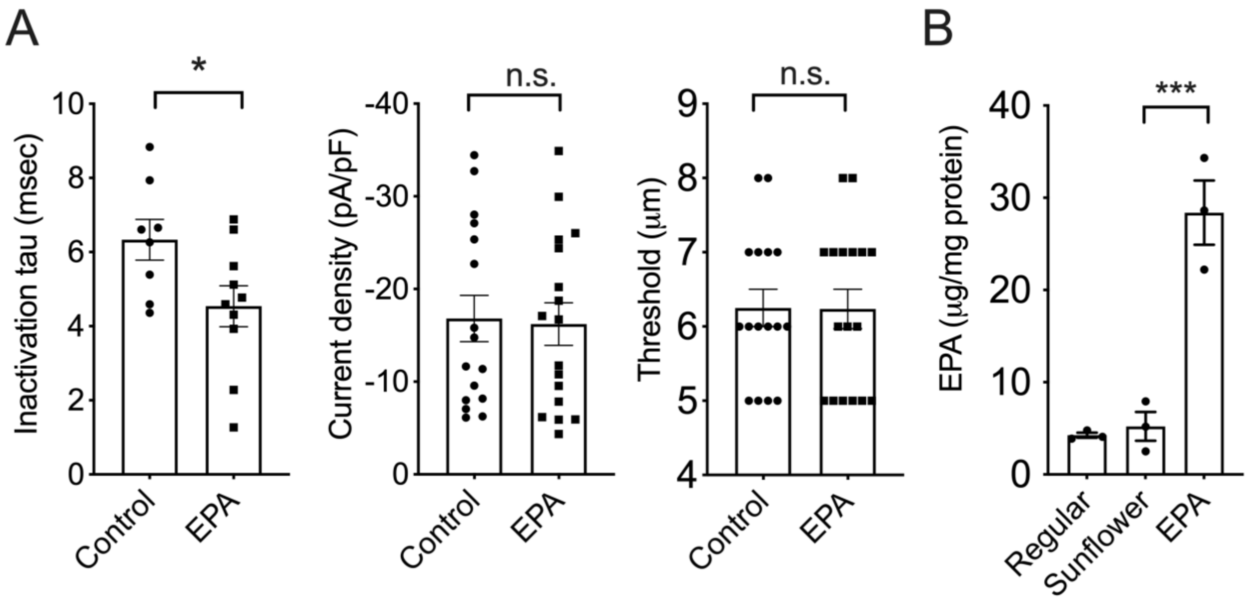

Fig. S5. (C) Whole cell recordings from wild type mice. EPA decreases the inactivation time constant of rapidly inactivating PIEZO2-dependent currents in DRG neurons (left) but has no effects on MA current magnitudes (I_{max}, middle) and apparent thresholds (right). (D) EPA membrane content of DRG from mice fed with standard, sunflower, or menhaden oil (w-3 EPA) enriched diets, as determined by LC-MS (Unpaired *t* test with Welch correction).
